# A Simplified non-invasive model based on albumin, bilirubin, and platelet count for predicting high-risk varices in compensated cirrhosis

**DOI:** 10.3389/fmed.2026.1865640

**Published:** 2026-07-23

**Authors:** Zhihui Duan, Xiaofang Sun

**Affiliations:** Endoscopy Center, Xingtai People’s Hospital, Xingtai, Hebei, China

**Keywords:** endoscopy, esophageal varices, liver cirrhosis, non-invasive prediction, portal hypertension

## Abstract

**Aim:**

Esophagogastroduodenoscopy (EGD) is the gold standard for detecting high-risk varices (HRV) in patients with liver cirrhosis, but is invasive and resource-intensive. The Albumin-Bilirubin-Platelet (ABP) model is non-invasive, but its performance requires validation in diverse populations. This study aimed to validate and refine the ABP criteria to predict HRV.

**Methods:**

This retrospective study included 474 patients with compensated cirrhosis, categorized into training (*n* = 332) and validation (*n* = 142) cohorts. All patients underwent EGD and laboratory evaluation. We evaluated the performance of the original and modified ABP (mABP) criteria, derived from the training cohort, to identify patients in whom screening EGD could be safely avoided.

**Results:**

The prevalences of HRV were 15.96% and 20.42% in the training and validation cohorts, respectively. In multivariate analysis of the training cohort, only platelet count was an independent predictor of HRV (*p* < 0.001). The original ABP criteria spared 21.83% of EGDs in the validation cohort with a 0% missed HRV rate. In the training cohort, the mABP criteria spared 51.51% of EGDs with a 2.34% missed HRV rate. The mABP criteria spared 58.45% of EGDs in the validation cohort, and the missed HRV rate was 4.82%.

**Conclusion:**

The original ABP criteria provide a simple and safe strategy for identifying low-risk patients with compensated cirrhosis. The modified ABP criteria substantially improve endoscopy avoidance while maintaining acceptable safety, offering a pragmatic alternative in settings where access to advanced diagnostic tools is limited.

## Introduction

Liver cirrhosis is the last stage of various chronic liver diseases and a major cause of morbidity and mortality worldwide (1). One of the most severe complications is portal hypertension, which leads to the formation of esophagogastric varices. High-risk varices (HRV), defined as esophagogastric varices with a diameter ≥5 mm or those with red wale marks, are associated with a significant risk of bleeding, with a mortality rate of 20% within 6 weeks of the first episode (2, 3).

Current recommendations of the American Association for the Study of Liver Diseases and the European Association for the Study of the Liver include screening esophagogastroduodenoscopy (EGD) for all patients newly diagnosed with cirrhosis to detect varices and initiate primary prophylaxis in the presence of HRV (4, 5). However, EGD is invasive, expensive, and associated with potential complications and patient discomfort. Furthermore, most patients with compensated cirrhosis do not have HRV at the initial screening, rendering the procedure unnecessary (6). This highlights the urgent need for accurate, reliable, and widely available non-invasive methods to stratify patients and identify those at low risk of having HRV, in whom screening EGD could be safely avoided or deferred.

Several such non-invasive predictors have been proposed in the past decade. The Baveno VI and subsequent Baveno VII consensus established criteria based on liver stiffness measurement (LSM) via transient elastography and platelet count (PLT) to predict the presence of HRV (4, 7). The Baveno VII criteria recommend that screening EGD can be safely avoided in patients with compensated advanced chronic liver disease with an LSM < 15 kPa and a PLT > 150 × 10^9^/L, as they have a low risk (<5%) risk of varices that require treatment. While effective, these criteria require access to transient elastography, which is not universally available, particularly in resource-limited settings.

To overcome this limitation, researchers have focused on models using routine laboratory parameters. Simple markers such as PLT (8), spleen diameter (8), and liver function test results (e.g., albumin and bilirubin levels) (9–15) have been incorporated into various predictive scores (9–15). Recently, a simple model with easy to examine parameters such as serum albumin and total bilirubin levels and PLT (the ABP criteria) was proposed to screening patients in whom endoscopy can be avoided, regardless of etiology (13, 14). However, the performance of the ABP criteria has not been validated in China.

The primary aim of this study was to validate and refine the ABP criteria in a large, independent cohort of patients with compensated cirrhosis.

## Materials and methods

This retrospective, cross-sectional single-center study included patients diagnosed with liver cirrhosis who underwent EGD for variceal screening between January 2018 and December 2024. Patients were divided into two cohorts using the split-sample method.

The inclusion criteria were as follows: (1) age ≥ 18 years; (2) a diagnosis of cirrhosis based on liver biopsy or a combination of clinical, laboratory, and imaging findings; (3) compensated cirrhosis, defined as Child-Pugh class A or B without decompensation events; and (4) availability of EGD and laboratory results within 6 months of each other.

The exclusion criteria were as follows: (1) history of variceal bleeding; (2) history of treatment for portal hypertension, such as beta-blockers, endoscopic variceal ligation, or transjugular intrahepatic portosystemic shunt; (3) presence of hepatocellular carcinoma or other malignancies; (4) portal or splenic vein thrombosis; (5) use of anticoagulant or antiplatelet drugs that could affect platelet count; and (6) incomplete clinical or laboratory data.

The study was conducted in accordance with the Declaration of Helsinki, and the protocol was approved by the Institutional Review Board (IRB) (Approval Number: 2024[055], approval date: 2024 May 14). The requirement for obtaining informed consent was waived by the IRB due to the retrospective nature of the study.

Demographic, clinical, and laboratory data were extracted from electronic medical records. The collected data included age, sex, body mass index, etiology of liver disease, and standard laboratory parameters: PLT; albumin (ALB), total bilirubin (Tbil), alanine aminotransferase, and aspartate aminotransferase levels; prothrombin time, and international normalized ratio. For patients who underwent transient elastography (FibroScan^®^), LSM and controlled attenuation parameter values were also recorded.

All endoscopic examinations were performed by experienced endoscopists. Esophageal varices were graded according to their size as small (F1, minimally elevated veins), medium (F2, tortuous veins occupying less than one-third of the esophageal lumen), or large (F3, occupying more than one-third of the lumen). The presence of red wale marks was also noted. HRV were defined as varices with a diameter ≥5 mm or any varices with red wale marks (7).

### Predictive criteria

We evaluated two sets of criteria based on the ABP model:

Original ABP Criteria: Based on the previously published literature (13): ALB concentration >40 g/L (13) was used as the standard criterion for initial validation. TB < 22 μmol/L and PLT > 114000/μL were recorded.

Modified ABP (mABP) Criteria: The cut-off values for ALB, TB, and PLT were optimized using the Classification and Regression Tree (CART) model analysis in the training cohort to maximize the number of patients in whom EGDs could be avoided while maintaining a low missed HRV rate. This study was a retrospective analysis based on a training cohort (*n* = 332) and a validation cohort (*n* = 142). In the training cohort, 53 patients (16.0%) were HRV-positive (HRV = 1) and 279 patients (84.0%) were HRV-negative (HRV = 0). In the validation cohort, 29 patients (20.4%) were HRV-positive and 113 patients (79.6%) were HRV-negative. Three non-invasive laboratory variables were included as predictors: PLT, ALB, and TB. A CART algorithm was used to construct the classification model, with PLT, ALB, and TB as independent variables and the presence of HRV (binary outcome) as the dependent variable. The model parameters were set as follows: Split criterion: Gini impurity, Gini = 1−Σ(p_*i*_^2^), where p_*i*_ represents the proportion of class i samples in a given node; Maximum tree depth (max depth): 3; Minimum number of samples per leaf node (minimum samples leaf): 10; Minimum number of samples required to split an internal node (minimum samples split): 20; Complexity parameter/pruning strategy: 10-fold cross-validation combined with the 1-standard error (1-SE) rule.

The results indicated that PLT was the most important splitting variable, with a root node threshold of PLT ≤ 95.5 (×10^9^/L).

### Threshold optimization strategy for the modified ABP rule

A systematic grid search was performed to identify the optimal threshold combination based on CART-derived splitting points, under the following constraints:

(1) Primary constraint: missed HRV rate < 5%
(2) Optimization objective: maximize the rate of spared endoscopy (EGD)

Search ranges:

(1) PLT: 85–119 (step size = 1)
(2) ALB: 32.0–39.5 (step size = 0.5)
(3) TB: 10–124 (step size = 2).

Additionally, candidate thresholds covering the 5th–90th percentiles of each variable were incorporated to ensure comprehensive coverage of the data distribution. All combinations meeting the constraint in the training cohort were further validated in the validation cohort.

### Model evaluation metrics

Esophagogastroduodenoscopy spared rate = (number of patients meeting ABP criteria ÷ total number of patients) × 100%

Missed HRV rate = (number of HRV cases among patients meeting ABP criteria ÷ total number of patients meeting ABP criteria) × 100%

Sensitivity = (number of HRV patients not meeting ABP criteria ÷ total number of HRV patients) × 100%

Specificity = (number of non-HRV patients meeting ABP criteria ÷ total number of non-HRV patients) × 100%

Negative predictive value (NPV) = (number of non-HRV patients meeting ABP criteria ÷ total number of patients meeting ABP criteria) × 100%

Positive predictive value (PPV) = (number of HRV patients not meeting ABP criteria ÷ total number of patients not meeting ABP criteria) × 100%.

### Statistical analysis

Continuous variables were presented as mean (standard deviation) or median (interquartile range) and compared using the Student’s *t*-test or Mann-Whitney U test, as appropriate. Categorical variables were presented as numbers (percentages) and compared using the chi-square test or Fisher’s exact test.

In the training cohort, a univariate logistic regression analysis was performed to identify factors associated with the presence of HRV. Variables with a *p*-value < 0.10 in the univariate analysis were included in a multivariate logistic regression model using a forward stepwise method to identify independent predictors of HRV. Odds ratios and their 95% confidence intervals were calculated.

To optimize the ABP criteria, for maximizing the number of patients eligible for these criteria (eligibility for the ABP criteria indicates that such patients can be exempted from gastroscopic screening) while ensuring that the percentage of HRV misdiagnosis among eligible patients is <5%, we adopted the Classification and Regression Tree (CART) model to identify the optimal cutoff values for PLT, Tbil concentration, and serum ALB concentration included in the ABP criteria. CART model: Gini impurity, max depth = 3, min samples leaf = 10, min samples split = 20, 10-fold cross-validation.

We also calculated sensitivity (true positive rate), specificity (true negative rate), false positive rate, and false negative rate. A two-sided *p*-value < 0.05 was considered statistically significant. All statistical analyses were performed using SPSS Statistics for Windows, Version 25.0 (IBM Corp., Armonk, NY), R version 4.1.2 and Python 3.11.

## Results

### Patient characteristics

A total of 474 patients met the inclusion criteria and were included in the final analysis. The training cohort consisted of 332 patients, and the validation cohort consisted of 142 patients. The baseline demographic, clinical, and laboratory characteristics of both cohorts are summarized in Table 1.

**TABLE 1:** Patients’s baseline characteristics.

Variables	Training cohort (*n* = 332)	Validation cohort (*n* = 142)
Age, years mean ± SD	52.67 ± 12.47	54.01 ± 11.55
Male *n* (%)	205 (61.75)	97 (68.31)
BMI Kg/m^2^ (median, IQR)	25.27 (22.53–27.98)	24.38 (22.66–26.71)
Etiology n (%)		
HBV	176 (53.01)	89 (62.68)
HCV	5 (1.51)	6 (4.23)
PBC/PSC	16 (4.82)	8 (5.63)
AIH	8 (2.41)	0 (0)
NAFLD	5 (1.51)	3 (2.11)
Fatty liver disease	2 (0.70)	5 (3.52)
Cryptogenic	112 (33.73)	29 (20.42)
Child-Pugh A	284(85.5)	124 (87.32)
Child-Pugh B	48 (14.5)	18 (12.68)
Platelet count (×10^9^/L)	112(80.25–164.0)	115(87.5–159.75)
Albumin (g/L)	41.0(37.03–44.30)	40.05 (36.58–43.70)
Total bilirubin (μMol/L)	18.95(13.43–31.10)	18.10 (13.53–26.10)
ALT (IU/L)	33.75(22.63–53.95)	37.0(21.95–55.95)
AST (IU/L)	36.10(24.53–59.28)	33.1(23.78–52.28)
PT	12.50(11.60–13.60)	12.20(11.60–13.10)
INR	1.15(1.07–1.25)	1.13(1.06–1.20)
LSM, kPa	18.30(13.98–29.13)	18.20(11.68–23.85)
CAP, dB/m	219.5(194.75–260.5)	218.0(190.75–257.75)
Esophageal varices, (%)	96 (28.92)	46 (32.39)
HRV, (%)	53(15.96)	29(20.42)

ALT, alanine aminotransferase; AST, aspartate aminotransferase; BMI, body mass index; IQR, interquartile range; HBV, hepatitis B virus; HCV, hepatitis C virus; PBC, primary biliary cirrhosis; PSC, primary sclerosing cholangitis; AIH, autoimmune hepatitis; NAFLD, non-alcoholic fatty liver disease; LSM, liver stiffness measurement; CAP, controlled attenuation parameter; HRV, high-risk varices; PT, prothrombin time; INR, international normalized ratio.

### Independent predictors of HRV

The results of logistic regression analyses for predictors of HRV in the training cohort are shown in Table 2. In the multivariate analysis, only PLT was significantly associated with the presence of HRV.

**TABLE 2:** Predictors of HRV in training cohort by univariate analysis and multivariate analysis, respectively.

Variable	Univariate analyses			Multivariate analyses	
	HRV + (*N* = 53)	HRV - (*N* = 279)	*P*-value	OR (95% CI)	*P*-value
Age (years)	51.36 ± 10.36	52.92 ± 12.83	0.405	–	–
Male *n* (%)	33 (62.26)	172 (61.65)	0.933	–	–
BMI (mean, SD)	27.14 ± 3.29	25.06 ± 3.92	0.006	1.133 (0.963–1.334)	0.133
Child-Pugh A *n* (%)	44 (83.02)	240 (86.02)	0.569	–	–
ALT, U/L	32.8 (24.0–53.5)	34.3 (22.1–54.3)	0.898	–	–
AST, U/L	38.9 (28.5–66.9)	35.6 (24–57)	0.209	–	–
Albumin, g/L	39.43 ± 4.82	40.63 ± 5.18	0.118	1.031 (0.962–1.106)	0.386
Total bilirubin, umol/L	22.4 (17.15–37.8)	18.6 (13–29.4)	0.024	1.004 (0.995–1.014)	0.359
PT	13.2 (12.4–14.95)	12.4 (11.5–13.33)	<0.001	0.952 (0.653–1.389)	0.797
INR	1.23 (1.13–1.36)	1.14 (1.06–1.23)	<0.001	15.144 (0.156–1468.23)	0.244
Platelet count, ×10^9^/L	77 (57–90.5)	126 (88–172)	<0.001	0.970 (0.959–0.981)	**<0.001**
LSM, kPa	17.1 (15.3–25.4)	18.4 (13.8–29.6)	0.950	–	–
CAP, dB/m	245 (179–274)	219 (196–260)	0.717	–	–

BMI, body mass index; LSM, liver stiffness measurement; CAP, controlled attenuation parameter; ALT, alanine aminotransferase; AST, aspartate aminotransferase; PT, prothrombin time; INR, international normalized ratio; HRV, high-risk varices. Bold value means *P* < 0.001.

### mABP criteria

The resulting optimal ABP cutoff values were as follows: PLT > 94 × 10^9^/L, ALB concentration >35 g/L, and Tbil concentration <100 μmol/L.

### Performance of the predictive criteria

**FIGURE 1:**
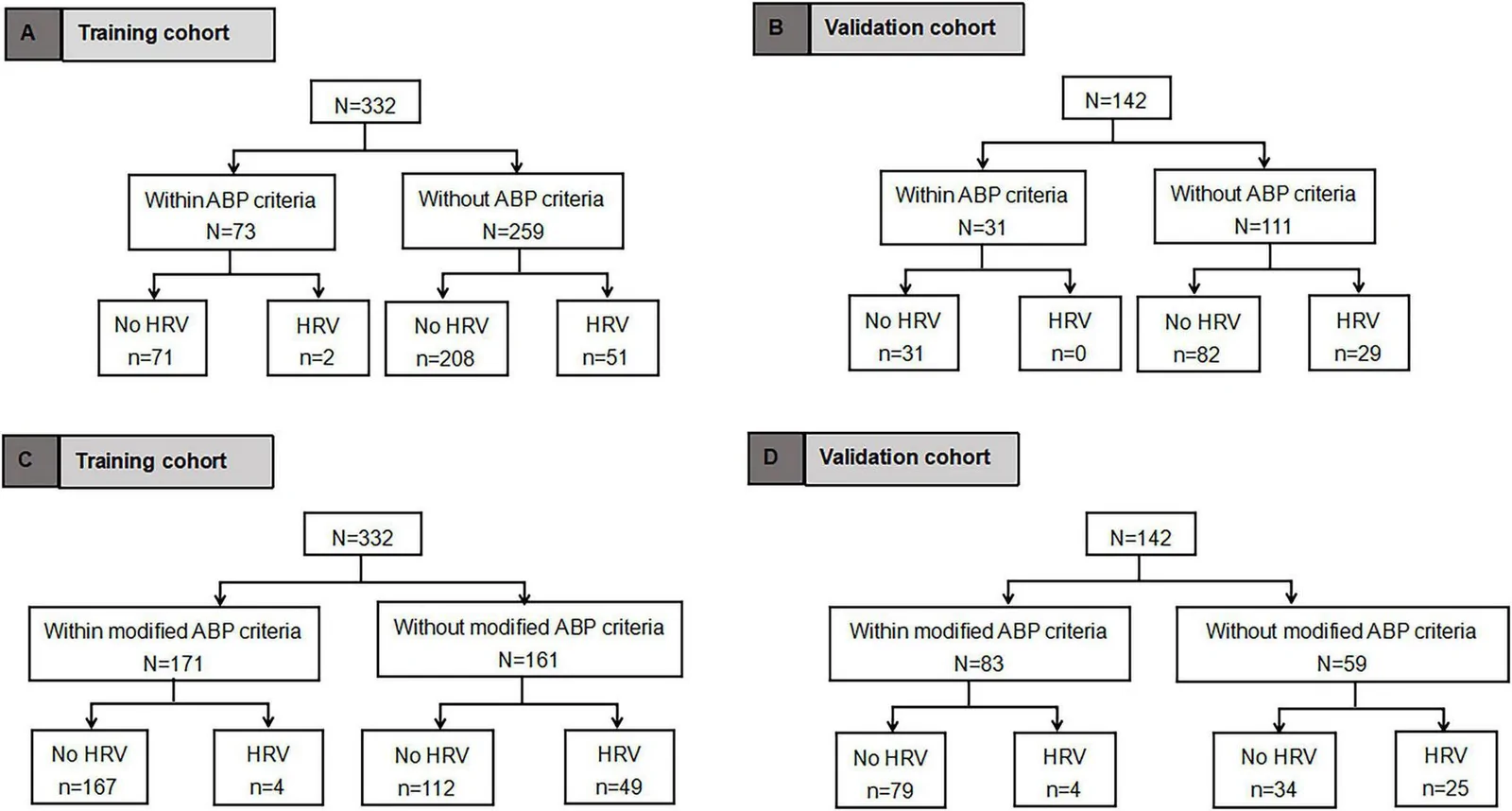
Diagnostic flow of HRV by the ABP criteria and modified ABP criteria in training cohort and validation cohort. **(A,B)** Depict the diagnostic performance of the ABP criteria in the training and validation cohorts, respectively, while **(C,D)** show that of the modified ABP criteria. HRV, high-risk varices; ABP, Albumin-Bilirubin-Platelet; N, number.

**FIGURE 2:**
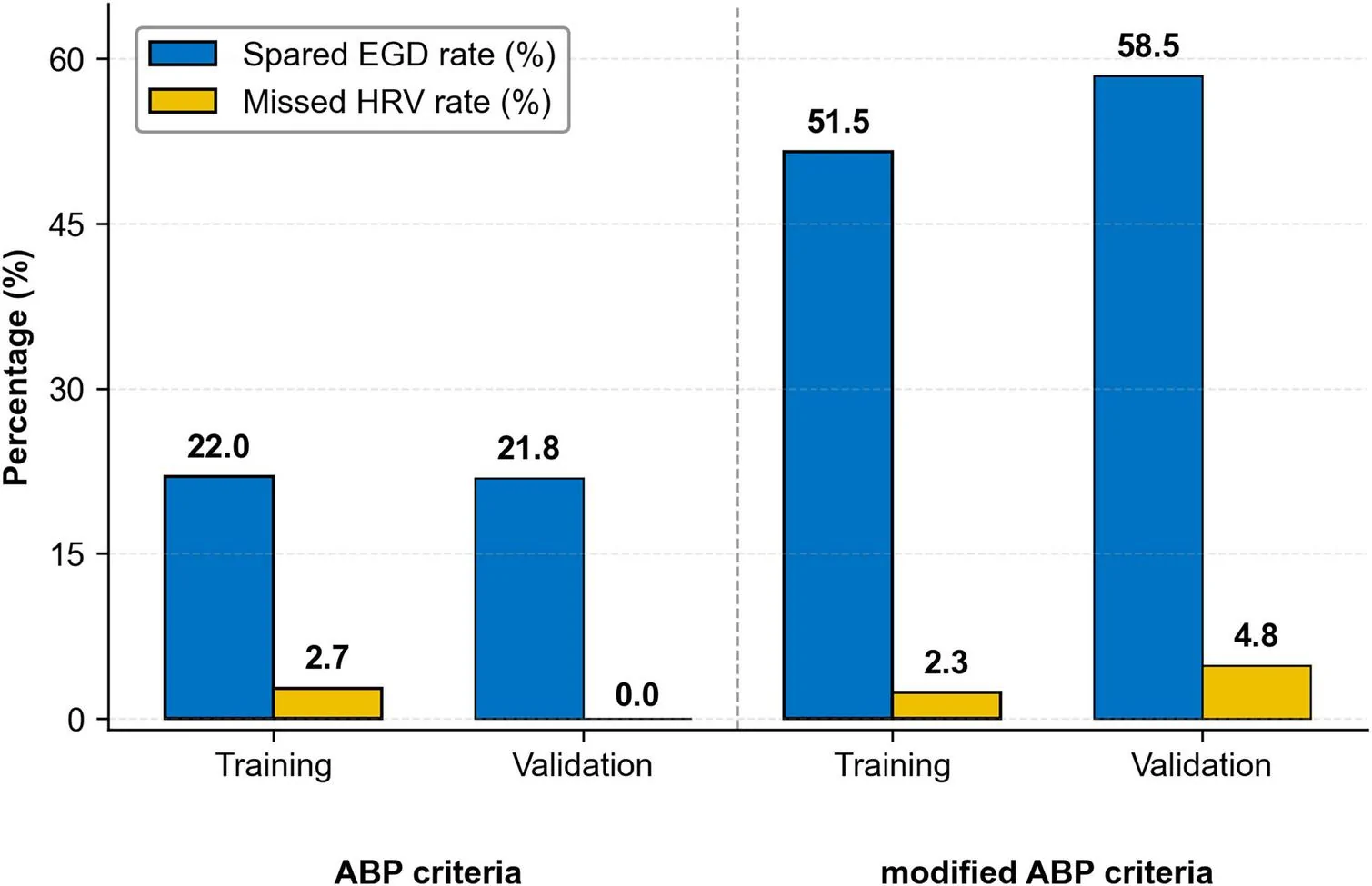
Performance of ABP criteria (ALB > 40 g/L and TB < 22 μmol/L and PLT > 114 × 10^9^/L) and modified ABP criteria (ALB > 35 g/L, TB < 100 μmol/L and PLT > 94 × 10^9^/L) to avoid screening endoscopy in patients with liver cirrhosis and the high-risk varices missed rate. EGD, esophagogastroduodenoscopy; ABP, Albumin-Bilirubin-Platelet; HRV, high-risk varices.

The performances of the original ABP and the newly derived mABP criteria were evaluated in both cohorts. The results are detailed in Table 3 and represented in Figures 1, 2.

**TABLE 3:** Performance of ABP and modified ABP criteria to rule out high-risk varices in patients with liver cirrhosis.

Cohort	Criteria	*N*	HRV +	EGD spared rate	Missed HRV rate	Sen	Spe	NPV	PPV
Training	ABP	332	53	21.99	2.74	96.23	25.45	97.26	19.69
Validation	ABP	142	29	21.83	0.00	100.00	27.43	100.0	26.13
Training	mABP	332	53	51.51	2.34	92.45	59.86	97.66	30.43
Validation	mABP	142	29	58.45	4.82	86.21	69.91	95.18	42.37

*N*, number; HRV, high-risk varices; Sen, sensitivity; Spe, specificity; PPV, positive predictive value; NPV, negative; EGD, esophagogastroduodenoscopy; HRV, high-risk varices.

In the training cohort, the ABP criteria correctly identified 71 patients as low-risk, avoiding 21.99% (73/332) of EGDs. However, 2 patients with HRV were missed, resulting in a missed HRV rate of 2.74% (2/73). The mABP criteria identified 171 patients as low-risk, sparing a much larger proportion of EGDs at 51.51% (171/332). This came at the cost of missing 4 patients with HRV, for a missed HRV rate of 2.34% (4/171). In the validation cohort, the ABP criteria spared 21.83% (31/142) of EGDs. Impressively, it did not miss any patients with HRV, achieving a missed HRV rate of 0%. The mABP criteria demonstrated superior utility in reducing endoscopic procedures, sparing 58.45% (83/142) of EGDs. Among these 83 patients classified as low-risk, 4 had HRV, corresponding to a missed HRV rate of 4.82%.

## Discussion

In the present study, we validated and refined a simple, non-invasive model using only routine laboratory test results—ALB and TB and PLT (ABP criteria)—to predict the presence of HRV in patients with compensated cirrhosis. The ABP criteria exhibited a stable performance in both training and validation cohorts: approximately 21% of EGD procedures were spared across both cohorts, while the missed HRV rate was maintained below 5%. Notably, the EGD-sparing rate with the ABP criteria was lower than the range (31.5%–39.2%) reported in the literature (13–15). However, after modification of the ABP criteria, the EGD-sparing rate increased to over 50% in our cohorts—suggesting that the ABP criteria require optimization for application in the Chinese population. Our findings demonstrate that such a model can effectively stratify patients, potentially sparing a substantial number of unnecessary screening endoscopies. The mABP criteria, in particular, permitted avoiding EGDs in nearly half of the patients in the validation cohort, a significant improvement over original ABP criteria.

The need for non-invasive tools to replace or supplement screening EGDs is well-established. The Baveno VI and VII criteria have set a benchmark, emphasizing a missed HRV rate of less than 5% (4, 7). The decision to adopt such a strategy depends on local healthcare resources, patient preferences, and the perceived risk of a missed diagnosis versus the risks and costs of universal screening.

When comparing our results to published literature, the performance of the mABP criteria is promising. The original ABP criteria spared about 21% of endoscopies with a <5% HRV missed rate in our cohorts. Our mABP model spared more than twice that number. Other models based on PLT/spleen diameter ratio have shown a variable performance and often require specific imaging, which may not be standardized (8). The ABP model relies on three of the most robust and reproducible laboratory parameters available. The ALIBI-PLT was developed as a non-invasive tool to predict advanced fibrosis in patients with chronic liver disease (16, 17). The ALBI score is promising in predicting HRV and esophageal varices in patients with cirrhosis (18). Furthermore, the Early Prediction of Decompensation Score, which is based on three biomarkers (ABP), enables the prediction of decompensation risk in patients with liver cirrhosis (19). Our research findings serve as a supplement and validation to the several previous studies on ALB, bilirubin and PLT (16–19).

This study has several strengths. First, it includes a reasonably large sample size with a dedicated, temporally distinct validation cohort, which enhances the robustness of our findings. Second, the study population is representative of cirrhosis etiologies in many parts of Asia, with HBV being predominant.

However, several limitations must be acknowledged. First, this was a retrospective, single-center study, which may have introduced selection bias and limited the generalizability. Second, while we confirmed the importance of LSM, we intentionally developed a model that did not require it, aiming for a broader applicability. The performance might be further improved by incorporating LSM, but at the cost of simplicity and accessibility.

In conclusion, our study validates the utility of a simple model based on ALB, TB and PLT for non-invasive screening for HRV in patients with compensated cirrhosis. The mABP criteria can identify a large subgroup of patients at a low risk for HRV, safely sparing them from unnecessary endoscopy and reducing the burden on healthcare systems. This simplified approach may serve as a practical alternative to elastography-based strategies, particularly in healthcare settings where access to advanced diagnostic tools is limited. Future prospective, multi-center studies are warranted to confirm these findings and establish the long-term safety of this screening strategy in diverse patient populations.

## Lay summary

Esophagogastroduodenoscopy (EGD) is the gold standard for detecting high-risk varices (HRV) in compensated cirrhosis but is invasive. This retrospective study validated the non-invasive Albumin Bilirubin Platelet (ABP) model in 474 patients. Standard ABP safely spared 21.83% of EGDs with 0% missed HRV. A modified ABP (mABP) model (PLT > 94 × 10^9^/L, ALB > 35 g/L, and TB < 100 μmol/L) reduced unnecessary endoscopies by nearly 50% with HRV missed rate less than 5%.

## Data Availability

The original contributions presented in this study are included in this article, further inquiries can be directed to the corresponding author.
