# Correction: A simplified non-invasive model based on albumin, bilirubin, and platelet count for predicting high-risk varices in compensated cirrhosis

**DOI:** 10.3389/fmed.2026.1951053

**Published:** 2026-09-22

**Authors:** Zhihui Duan, Xiaofang Sun

**Affiliations:** Endoscopy Center, Xingtai People’s Hospital, Xingtai, Hebei, China

**Keywords:** endoscopy, esophageal varices, liver cirrhosis, non-invasive prediction, portal hypertension

There was a mistake in Table 1 as published. In the table footnote, the abbreviation for IQR was incorrectly given as “IQR, interquartile ratio”. This has been corrected to “IQR, interquartile range”. The corrected footnote reads:

ALT, alanine aminotransferase; AST, aspartate aminotransferase; BMI, body mass index; IQR, interquartile range; HBV, hepatitis B virus; HCV, hepatitis C virus; PBC, primary biliary cirrhosis; PSC, primary sclerosing cholangitis; AIH, autoimmune hepatitis; NAFLD, non-alcoholic fatty liver disease; LSM, liver stiffness measurement; CAP, controlled attenuation parameter; HRV, high-risk varices; PT, prothrombin time; INR, international normalized ratio.

There was a mistake in Table 3 as published. In the “Cohort” column, “Validating” was incorrectly given instead of “Validation”. This has been corrected to read: “Validation”.

In the published article, there was an error in the **Ethics statement**. The date of ethical approval was erroneously given as “May 13, 2024”. The correct date is “May 14, 2024”. The correct statement appears below:

“The studies involving humans were approved by the ethics committee of Xingtai People's Hospital on May 14, 2024 (approval no. 2024055). As it did not involve patients’ privacy, diagnosis, or treatment, informed consent was waived by the ethics committee of Xingtai People's Hospital. This study adhered to the Declaration of Helsinki. The studies were conducted in accordance with the local legislation and institutional requirements. Written informed consent for participation was not required from the participants or the participants’ legal guardians/next of kin in accordance with the national legislation and institutional requirements.”

In the published article, there was an error in the **Lay summary**. The mathematical comparison symbols in the threshold formula failed to display correctly.

A correction has been made to the section **Lay summary**, paragraph 1:

“Esophagogastroduodenoscopy (EGD) is the gold standard for detecting high-risk varices (HRV) in compensated cirrhosis but is invasive. This retrospective study validated the non-invasive Albumin Bilirubin Platelet (ABP) model in 474 patients. Standard ABP safely spared 21.83% of EGDs with 0% missed HRV. A modified ABP (mABP) model (PLT > 94 × 10⁹/L, ALB > 35 g/L, and TB < 100 µmol/L) reduced unnecessary endoscopies by nearly 50% with HRV missed rate less than 5%.”

The original version of this article has been updated.

