# Supplementary material for: A Simplified non-invasive model based on albumin, bilirubin, and platelet count for predicting high-risk varices in compensated cirrhosis
Source: Front Med (Lausanne). 2026 Jul 23;13:1865640. doi: 10.3389/fmed.2026.1865640 (PMC13445703; doi:10.3389/fmed.2026.1865640)
Supplement: Supplementary file 1 [file Table_1.DOC]

### **Title: A Simplified Non-invasive Model Based on Albumin, Bilirubin, and Platelet Count for Predicting High-Risk Varices in Compensated Cirrhosis**

**Authors:Zhihui Duan^1^**^#^**,Xiaofang Sun^1^**

^#^corresponding authors:Zhihui Duan

**Supplementary Table 1. Performance of ABP and modified ABP criteria to rule out high-risk varices in patients with liver cirrhosis**

| **Cohort** | **Criteria type** | **Spared EGD** | **Missed HRV** | **Right positive** | **False positive** | **Right negative** | **False negative** |
| --- | --- | --- | --- | --- | --- | --- | --- |
| Traing cohort | ABP | 21.99% | 2.74% | 19.69% | 80.31% | 97.26% | 2.74% |
| Validation cohort | ABP | 21.83% | 0 | 18.02% | 73.87% | 100% | 0% |
| Traing cohort | modified ABP | 43.67% | 2.07% | 26.74% | 73.26% | 97.93% | 2.07% |
| Validation cohort | modified ABP | 49.30% | 5.71% | 34.72% | 58.33% | 94.29% | 5.71% |

**EGD:Esophagogastroduodenoscopy; HRV:high-risk varices**
